# An adult case of congenital duodenal diaphragm that was successfully treated by endoscopic resection using a grasping‐type scissor forceps

**DOI:** 10.1002/deo2.93

**Published:** 2022-02-02

**Authors:** Shin‐ichiro Fukuda, Kaoru Ichida, Yusuke Kitagawa, Kayoko Nakano, Chaen Tomohito, Daisuke Yoshimura, Toshiaki Ochiai, Eikichi Ihara, Yoshihiro Ogawa

**Affiliations:** ^1^ Depertment of Gastroenterology Saiseikai Fukuoka General Hospital Fukuoka Japan; ^2^ Department of Medicine and Bioregulatory Science Graduate School of Medical Sciences Kyushu University Fukuoka Japan

**Keywords:** congenital duodenal diaphragm, endoscopic resection, upper gastrointestinal bleeding, ClutchCutter

## Abstract

Congenital duodenal diaphragm (CDD) is a rare disease that is usually diagnosed in the neonatal period; however, it is sometimes diagnosed later in the adult period. A 39‐year‐old woman was referred to our hospital due to tarry stool and anemia. Emergent esophagogastroduodenoscopy (EGD) revealed an obstructing membranous structure with a small orifice in the second portion of the duodenum, together with dilatation of the bulbar part. The membranous structure was accompanied by a Dieulafoy‐like vessel on the backside, which was considered to have caused tarry stool and anemia. The Dieulafoy‐like vessel was successfully treated by endoscopic hemostasis. Based on the computed tomographic gastrography and barium duodenography findings, it was diagnosed as CDD. Later, endoscopic resection of the diaphragm was conducted by an endoscopic submucosal dissection (ESD)‐based procedure, with the use of an electrosurgical grasping‐type scissor forceps (ClutchCutter [CC]). There were no procedure‐related complications. The definite diagnosis of CDD was made based on the observation of typical structures in a pathological examination. This is the first case report of adult CDD that was successfully treated by endoscopic resection using ESD‐based techniques with a CC.

## INTRODUCTION

Congenital duodenal diaphragm (CDD) is a rare disease in which the duodenum, mostly the second part, is obstructed by a diaphragm‐like structure, which secondarily causes dilatation of the stomach and duodenal bulb. Patients with CDD usually start complaining of symptoms such as vomiting and abdominal distension in the neonatal or infant periods. The incidence of CDD is reported to be approximately one in 9000–40,000 live births.^1^ However, it is sometimes diagnosed later in the adult period. We herein report the case of a 39‐year‐old woman with CDD presenting upper gastrointestinal bleeding who was successfully treated by an endoscopic approach.

## CASE REPORT

A 39‐year‐old woman was emergently referred to our hospital due to repeated tarry stool with abdominal distension and regurgitation suspecting gastrointestinal bleeding. Her past medical history included frequent episodes of vomiting after meals during infancy. The vomiting symptoms disappeared when she was an elementary school student, while she sometimes suffered from abdominal distension and regurgitation after meals. Although she was nearly at the lower limit of the growth curve, no abnormality was pointed out. Her height, weight, and body mass index were 148 cm, 44.5 kg, and 20.3 kg/m^2^, respectively. Her hemoglobin level (8.1 g/dl) and serum iron level (36 μg/dl) at referral were lower than the lower limits of normal; however, her mean corpuscular volume (91.2 fl) was within the normal range, suggesting acute—but not chronic—gastrointestinal bleeding. She was hospitalized for further examinations.

**FIGURE 1:**
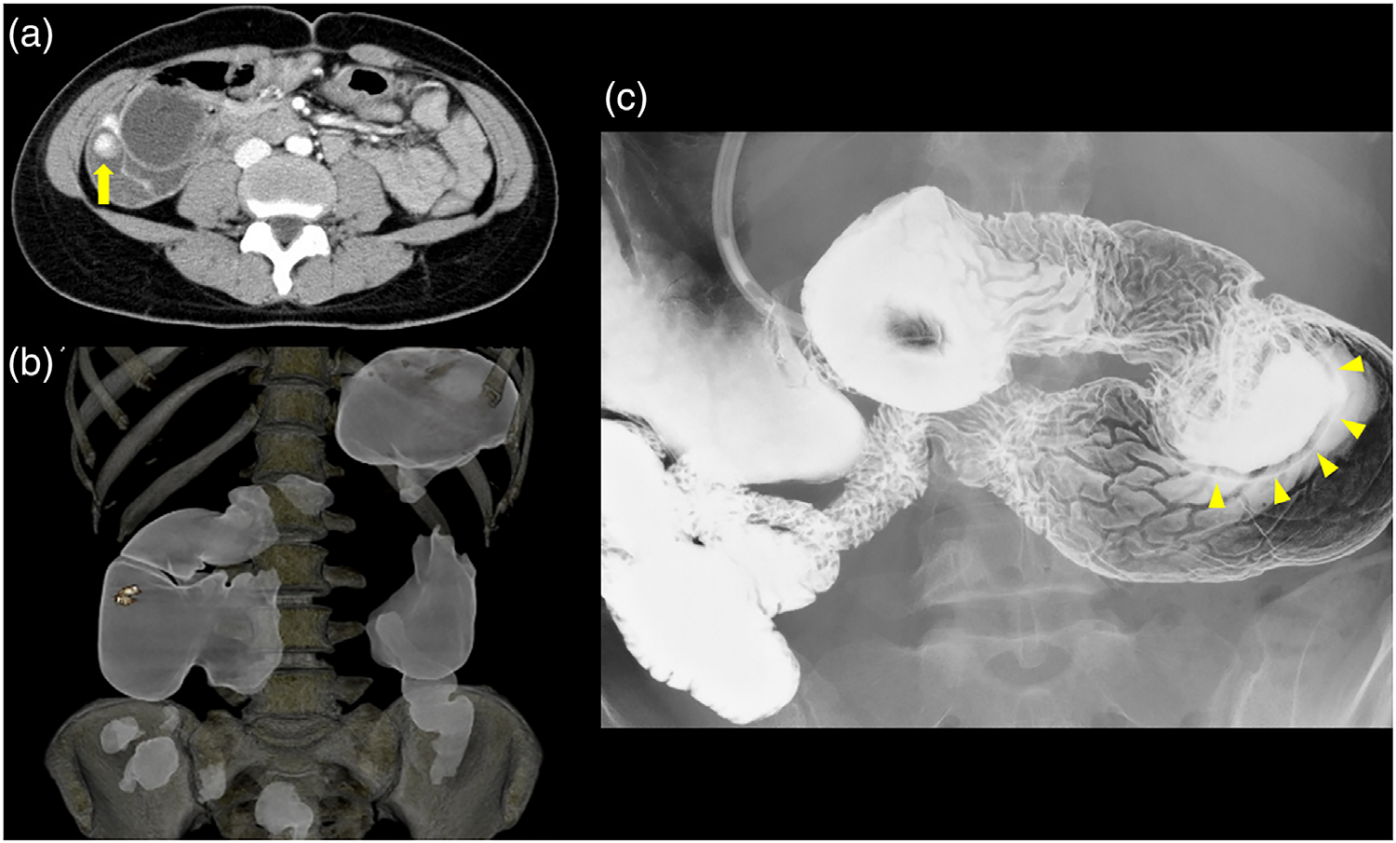
Radiographic examinations conducted for tarry stool and anemia associated with abdominal distension and regurgitation. (a) An abdominal computed tomography (CT) slice revealing dilatation of the duodenal bulb, and a membranous structure in the second part of the duodenum with contrast media extravasation (yellow arrow). (b) A representative 3D‐CT image showing the typical “double‐bubble sign”. (c) A representative barium duodenography image obtained with the patient in the prone position showing a membranous structure of approximately 3 mm in thickness arising from the second part of the duodenum (yellow arrowhead).

**FIGURE 2:**
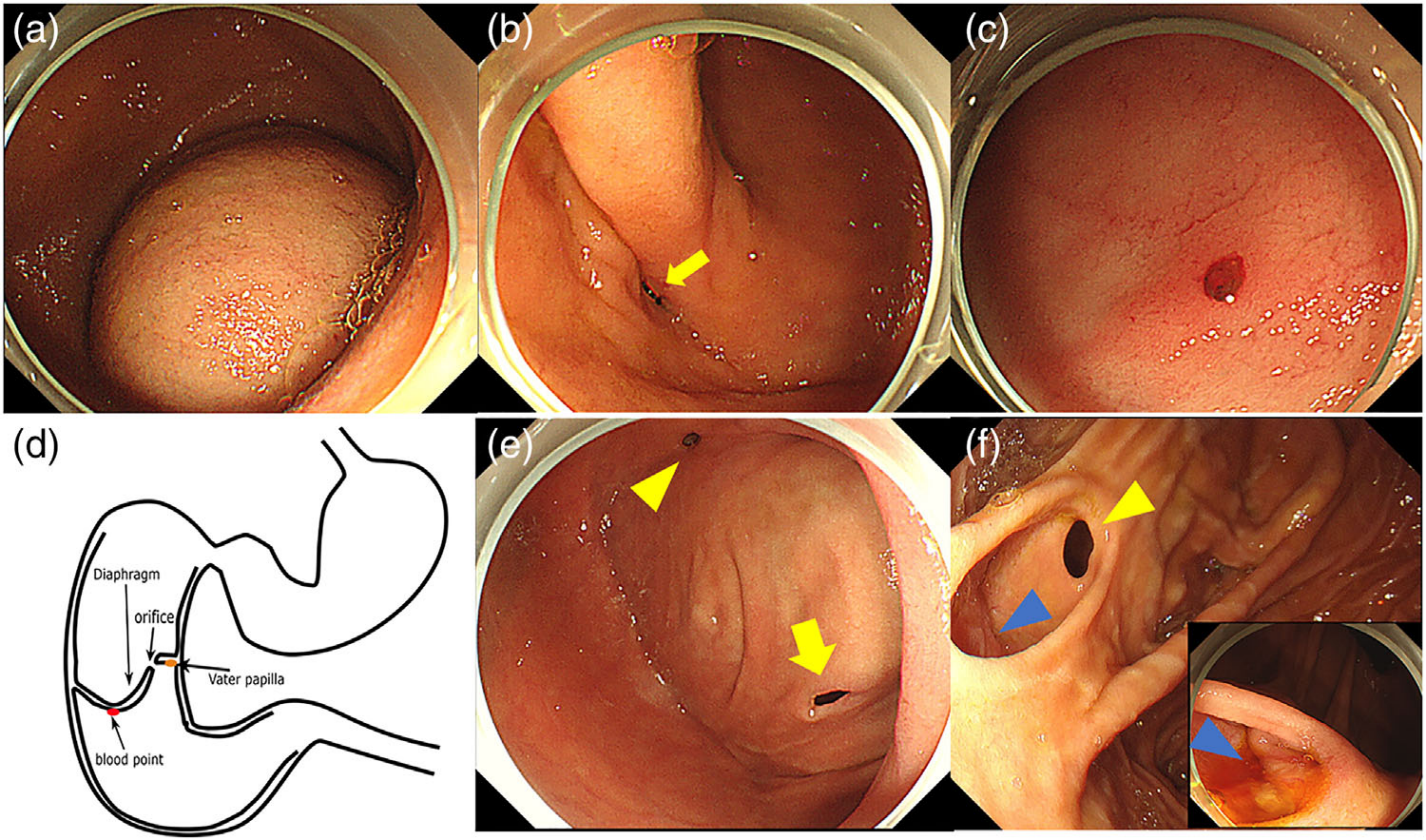
Endoscopic examinations. (a–c) Endoscopic examination to be conducted initially. Representative pictures showing a membranous structure protruding to the oral side on respiration in the second part of the duodenum (a), obstruction of the lumen with a membranous structure with a small orifice (yellow arrow) (b), and a retroflexed view obtained with a smaller caliber scope (Olympus PCF‐PQ260L) that passed through the small orifice (c). A Dieuafoy‐like blood vessel was observed on the backside of the membranous structure (c). (d) A schematic illustration of the anatomical structure of the duodenum in the present case. (e, f) Endoscopic examination to be conducted as second look examinations after endoscopic hemostasis. Representative pictures showing the newly created hole in the diaphragm where hemoclips had been placed (yellow arrow), as well as the original orifice (yellow arrowhead) (e), and a retroflexed view obtained with a smaller caliber scope (Olympus PCF‐PQ260L) that passed through the newly created hole (f). The papilla of Vater (blue arrowhead) was located on the anal side of the diaphragm, close to the original orifice (yellow arrowhead), where a close‐up view of the papilla of Vater was inserted (bottom right) (f).

Contrast‐enhanced computed tomography (CT) of the abdomen revealed dilatation of the duodenal bulb and a membranous structure in the second part of the duodenum from which the extravasation of the contrast agent was detected (Figure 1a). A typical “double‐bubble sign” consisting of gastric and proximal duodenal gas was seen on plain X‐ray and 3D‐CT of the abdomen (Figure 1b). Barium duodenography revealed a duodenal membranous structure of approximately 3 mm in thickness arising from the second part of the duodenum, which significantly delayed barium flow (Figure 1c). EGD revealed an obstructing membranous structure in the second part of the duodenum, together with massive dilatation of the bulbar part. The membranous structure, which moved up and down like a ‘wind‐sock’ with respiration (Figure 2a), had a narrow orifice of up to 10 mm in diameter (Figure 2b). Based on these findings, we made a diagnosis of CDD. Since no lesions that could cause bleeding were found on the oral side of the diaphragm, we attempted to introduce the scope through the orifice to evaluate the lumen on the anal side. We could not advance the normal caliber scope (Olympus GIF‐Q260J; diameter, 9.9 mm) through the narrow orifice, but managed to pass a smaller caliber scope (Olympus PCF‐PQ260L; diameter, 9.2 mm) through the orifice. A Dieulafoy‐like blood vessel with a blood clot was then found on the backside of the diaphragm (Figure 2c,d), and endoscopic hemostasis was achieved using hemoclips. Second look EGD was performed a few days after endoscopic hemostasis. Interestingly, we detected a small hole that had not been observed on initial EGD, but which had newly appeared on the diaphragm at the site of hemoclip placement (Figure 2e). The papilla of Vater was observed on the anal side, adjacent to the diaphragm, and close to the original orifice (Figure 2f). EGD also revealed long‐segment Barrett esophagus as a long‐term complication of gastroesophageal reflux disease (GERD). Her anemia was significantly improved up to 12.3 g/dl 1 month after hemostasis of the exposed vessel of the diaphragm.

**FIGURE 3:**
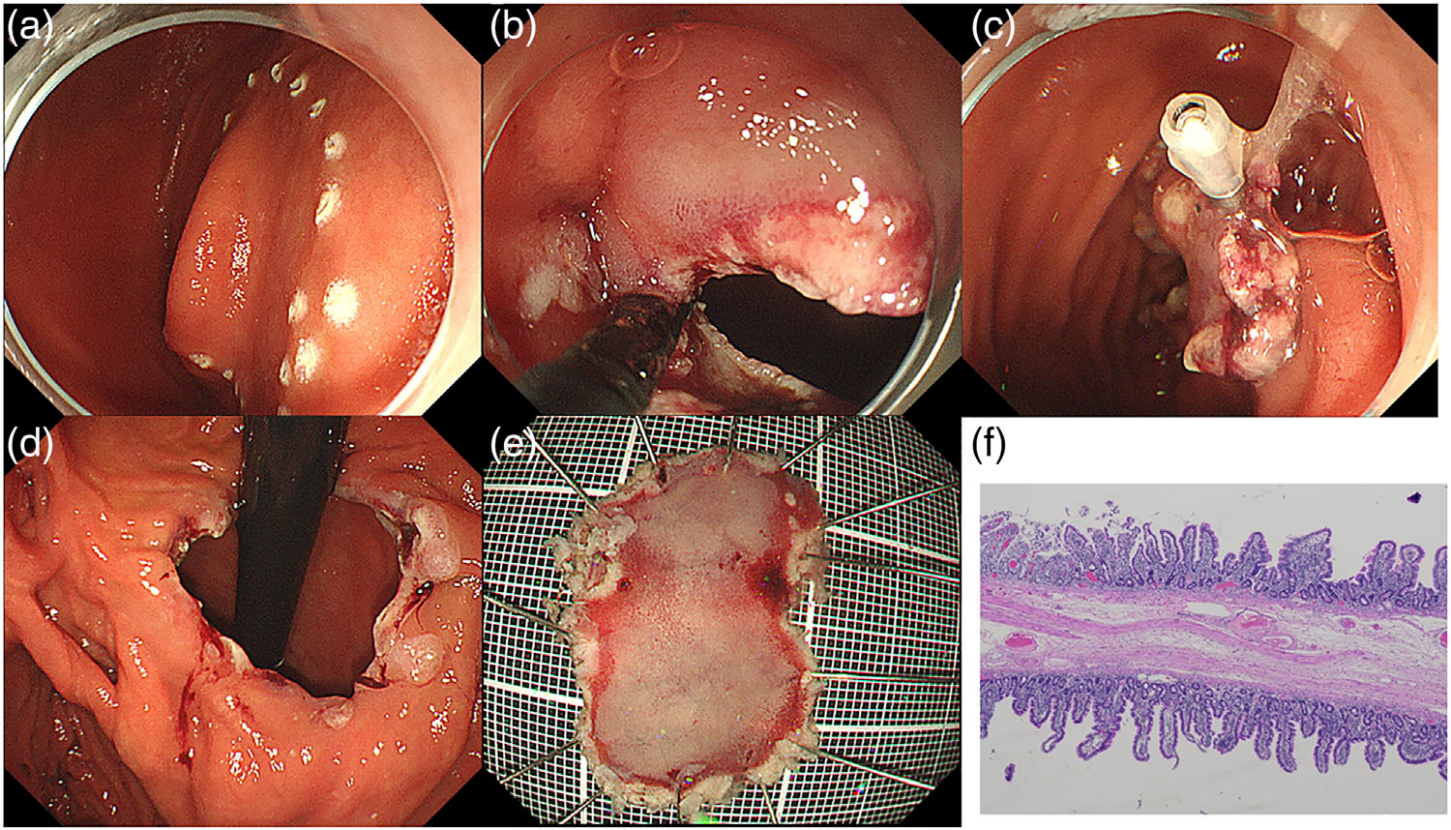
Endoscopic resection of the diaphragm using an endoscopic submucosal dissection (ESD)‐based procedure with a ClutchCutter. (a) The resection area was marked in an elliptical shape on the diaphragm using argon plasma coagulation. (b) A mural incision was started from the newly created hole using a ClutchCutter. (c, d) A clip‐with‐thread method was applied, and endoscopic resection of the target diaphragm was completed. (e) A macroscopic image of the resected specimen. (f) A pathological picture (HE) of the resected specimen showed that the diaphragm had a five‐layer structure consisting of the mucosa, muscularis mucosa, submucosa, muscularis mucosa, and mucosa.

Although she did not present weight loss after she was fully grown, she had been suffering from troublesome symptoms of abdominal distention and regurgitation after meals, which decreased her quality of life and labor productivity. Approximately 2 months after the initial EGD procedure, after discussing the endoscopic treatment of CDD with the patient, the diaphragm was resected by an endoscopic submucosal dissection (ESD)‐based procedure, using a grasping‐type scissor forceps (ClutchCutter [CC]; Fujifilm, Tokyo, Japan) approximately 2 months after initial EGD. In order to avoid damaging the papilla of Vater, the resection area was marked in an elliptical shape with argon plasma coagulation, with care taken to maintain a safe distance from the original orifice, which was very close to the papilla of Vater (Figure 2f). A mural incision was started from “the hole” that had been caused by the hemostatic procedure and proceeded along the spots marked on the incision line (Figure 3a,b). A series of mural incisions were further advanced, when required, using a clip‐with‐thread method to obtain appropriate traction (Figure 3c) until resection of the target area was completed (Figure 3d). There were no procedure‐related complications. Pathologically, the resected specimen had a five‐layer structure consisting of the mucosa, muscularis mucosa, submucosa, muscularis mucosa, and mucosa (Figure 3e,f), which was consistent with CDD. She was discharged 5 days after the endoscopic treatment without any problems. At the next visit, one month later, her abdominal distension and regurgitation had disappeared. EGD showed that the duodenal lumen created by endoscopic resection was still large enough to allow a normal caliber scope to smoothly pass through the diseased area. One year later, her body weight had increased from 44.5 to 47 kg.

## DISCUSSION

CDD is a rare congenital abnormality that occurs in approximately one in 9000–40,000 live births.^1^ It was first reported in 1845.^2^ A diaphragm is present in the duodenal lumen, where only a small orifice on the diaphragm allows content to pass towards the anal side. The congenital diaphragm is embryologically presumed to be a recanalization disorder of the fetal duodenum.^5^ The lumen of the intestinal tract of 30‐ to 60‐day‐old embryos has been shown to be obliterated by epithelial proliferation. Subsequently, the intestinal lumen was recanalized by vacuolization,^5^ where incomplete vacuolization led to the formation of a diaphragm. It has been reported that approximately half of the patients with CDD have an accompanying congenital anomaly of another organ system,^6^ including cardiac anomalies, malrotation of the gut, vertebral defect, or renal anomalies, which are sometimes associated with Down syndrome.^6^ However, in our case, the patient had no other congenital anomalies or relevant family history; thus, there was no clear hereditary predisposition.

Patients with CDD are most frequently diagnosed in the neonatal period; however, some patients (30%–35%) are diagnosed in the adult period, depending on the size of the orifice on the diaphragm.^3^ It has been reported that the proportion of patients who are diagnosed in the adult period will be high in comparison to the proportion of patients diagnosed in the neonatal period when the orifice is ≥8 mm in diameter,^4^ as it was in our case. Patients with CDD diagnosed in adulthood not only have accompanying symptoms of abdominal distension, vomiting, epigastric pain, and regurgitation but also growth disorder and severe GERD. Thus, this is the first report to describe a patient presenting upper gastrointestinal bleeding in the adult period.

Although surgery used to be a standard treatment for CDD, endoscopic treatment became an option after the first report of a case managed by an endoscopic incision in 1984.^7^ A search of MEDLINE (PubMed) for articles published the years 1984–2018 was conducted using the following keywords: “Congenital duodenal diaphragm”, “endoscopic treatment”. This search yielded 23 reports of cases that were managed by endoscopic treatment.^1^, ^8^, ^9^ One important point in the endoscopic treatment of CDD is to recognize the positional relationship of the diaphragm and papilla of Vater in order to avoid damaging the papilla of Vater during treatment. The diaphragm is most often close to the papilla of Vater; in two‐thirds of cases, the CDD was located on the anal side of the papilla of Vater, and in one‐third of cases, the CDD was located on the oral side.^10^ In our case, it was located on the oral side of the papilla of Vater, and the relative position of the papilla of Vater to the diaphragm was confirmed before endoscopic treatment.

Regarding the method of endoscopic treatment for CDD, various combinations of endoscopic treatment procedures (dilatation, ablation, incision, and resection) and endoscopic treatment devices (yttrium aluminum garnet laser, balloon, papillotome, insulated‐tip diathermic knife, needle knife, hook knife, polypectomy snare, and micro knife) have been applied (Table 1). As complications, postoperative bleeding was found in three of 23 cases (13%). In most cases, treatment was completed with a single endoscopic treatment procedure, but in some cases treated by balloon, dilation required repeated procedures or an additional endoscopic treatment procedure, such as mural incision.^9^ In contrast, there is also a report of a case with restenosis after a single mural incision.^8^ Endoscopic resection of the diaphragm with an ESD‐based procedure appears to be a more efficient method for overcoming duodenal obstruction. However, such procedures are associated with technical difficulties. In the process of completing endoscopic resection of the diaphragm, it will be difficult to conduct a series of mural incisions efficiently and safely using tip‐shaped devices because the diaphragm, with its thin structure, will be in a dangling state once the mural incision is started. An IT knife, which has an insulated tip at the distal end of a needle knife, in combination with the clip‐with‐thread method is a good option for this procedure. In this regard, however, the CC, an electrosurgical scissor forceps that can grasp and cut or coagulate tissue, will be much more suitable for endoscopic resection of the movable diaphragm with the thin structure. In our case, indeed, endoscopic resection of the diaphragm could be performed easily and safely with the CC in combination with the clip‐with‐thread method. This is the first reported case in which CDD was successfully treated by endoscopic resection using ESD‐based techniques with a CC.

**TABLE 1:** Reports on endoscopic treatment for congenital duodenal diaphragm

	**Author**	**Age**	**Sex**	**ET procedure**	**ET device**	**Complication**	**Subsequent clinical course**
1984	Gertsch	71 years	F	resection	YAG laser	no	good
1986	Kent	67 years	M	dilatation	balloon	no	poor
1986	Kent	81 years	M	incision	papillotome	no	additional ET
1989	Okamatsu	2 months	M	incision	HF knife	no	additional ET
1992	Yatsuka	2 months	M	incision	HF knife	no	good
1992	Ziegler	5 months	n.d.	ablation	laser	n.d.	surgery
1992	Kay	newborn	n.d.	ablation	laser	n.d.	good
1994	Kane	19 years	F	incision	papillotome	no	good
2000	Fujitomi	26 years	M	resection	polypectomy snare	bleeding	good
2005	Nose	3 years	M	resection	polypectomy snare	no	good
2005	Nose	1 year	M	incision	HF knife	no	good
2006	Suzuki	11 years	M	incision	HF knife, hook knife	no	good
2007	Akamatsu	28 years	M	resection	polypectomy snare, IT‐knife	bleeding	good
2007	Fukumura	1 year	F	incision	hook knife	n.d.	n.d.
2007	Otsu	2 months	F	dilatation, incision	balloon, papillotome	no	good
2008	Watanabe	1 year	M	incision	papillotome	no	surgery
2009	Ikenaga	3 years	F	dilatation, incision	balloon, hook knife	no	good
2010	Kunii	31 years	F	resection	polypectomy snare	bleeding	good
2010	Benes	30 years	F	incision	needle knife	no	good
2012	Bittencourt	9–12 months	F	dilatation, incision	balloon, needle knife	no	good
2015	Kong	2 years	M	dilatation	balloon	no	no
2015	Kong	19 years	F	incision	micro knife	no	no
2016	Poddar	9 years	M	dilatation	balloon	no	good
2016	Poddar	8 years	F	dilatation	balloon	no	good
2016	Poddar	2 years	F	dilatation	balloon	no	good
2021	This case	39 years	F	resection	ClutchCutter	no	good

## CONFLICT OF INTEREST

The authors declare that they have no conflict of interest.

## FUNDING INFORMATION

The authors received no financial support for this case report.
